# A case of impending paradoxical embolism

**DOI:** 10.1186/s44215-025-00229-y

**Published:** 2025-10-21

**Authors:** Hideaki Kanda, Kazuhisa Matsumoto, Akira Hiwatashi, Yoshiharu Soga

**Affiliations:** Department of Cardiovascular Surgery, Kagoshima University Graduate School of Medical and Dental Sciences, 8-35-1 Sakuragaoka, Kagoshima, 890-8520 Japan

**Keywords:** Impending paradoxical embolism, Pulmonary embolism, Patent foramen ovale

## Abstract

**Background:**

Impending paradoxical embolism (IPDE) is a rare condition characterized by the presence of a thrombus in a patent foramen ovale. We were able to image a thrombus lodged in a patent foramen ovale and remove the thrombus before it could result in arterial embolism.

**Case presentation:**

A 24-year-old woman was admitted to our hospital with chest pain and dyspnea. Pulmonary embolism was diagnosed via computed tomography. Additionally, transesophageal echocardiography revealed a thrombus lodged in a patent foramen ovale and fluttering in both atria, leading to a diagnosis of IPDE. Rather than incurring the risk associated with embolization by thrombolytic treatment, we elected to remove the incarcerated thrombus and close the patent foramen ovale under cardiopulmonary bypass.

**Conclusions:**

We successfully treated a case of IPDE by removing the thrombus under cardiopulmonary bypass, before arterial thrombosis could occur.

## Background

Impending paradoxical embolism (IPDE) is a rare condition characterized by the presence of a thrombus in a patent foramen ovale. Because IPDE can lead to cerebral infarction or other arterial embolism, early therapeutic intervention is required. We report a case of IPDE complicated by pulmonary embolism, wherein a thrombus was incarcerated in a patent foramen ovale and fluttering occurred in both atria. Surgical thrombectomy was performed before atrial embolism could occur.

## Case presentation

**Fig. 1:**
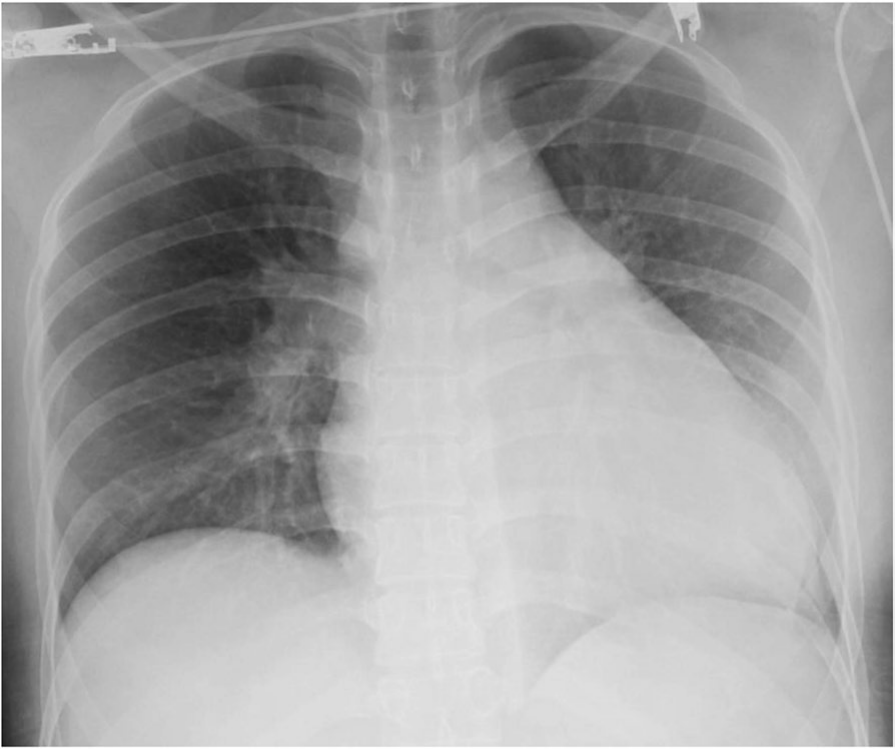
Preoperative chest radiograph showing remarkable enlargement of the cardiac shadow

**Fig. 2:**
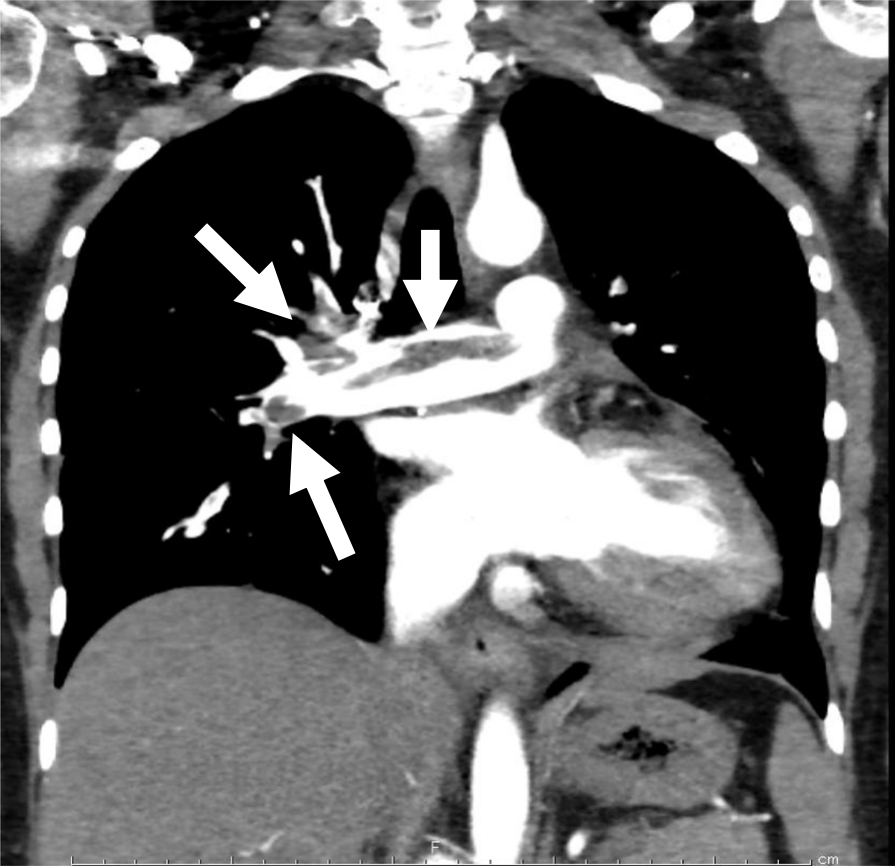
Computed tomography showing a thrombus (arrow) in the pulmonary artery

**Fig. 3:**
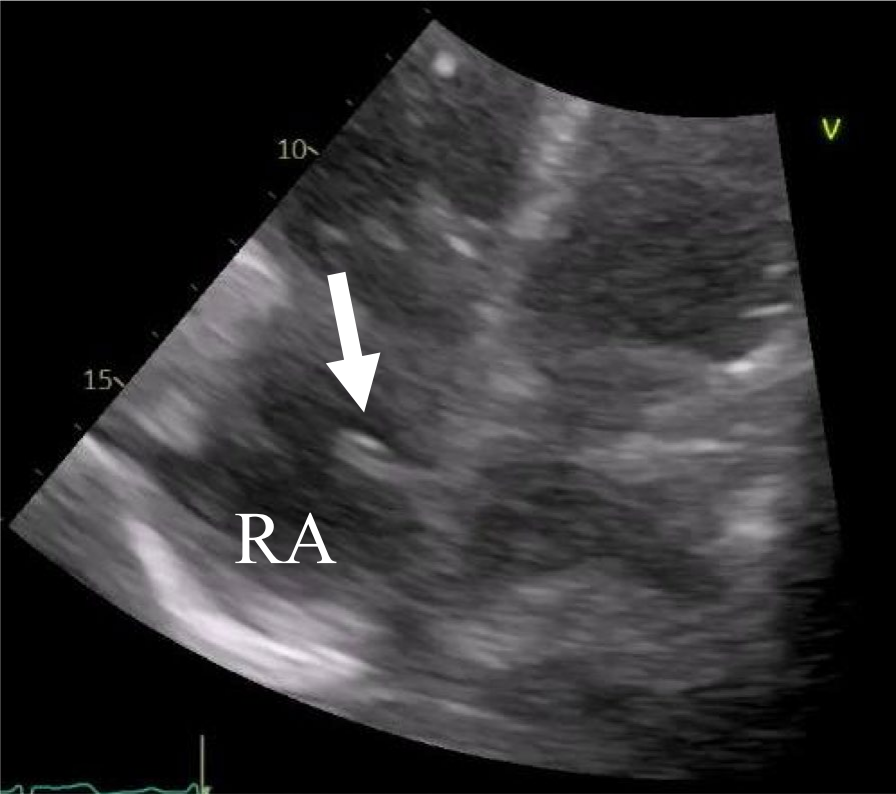
Transthoracic echocardiography showing a thrombus lodged in the foramen ovale (arrow: thrombus, RA: right atrium)

**Fig. 4:**
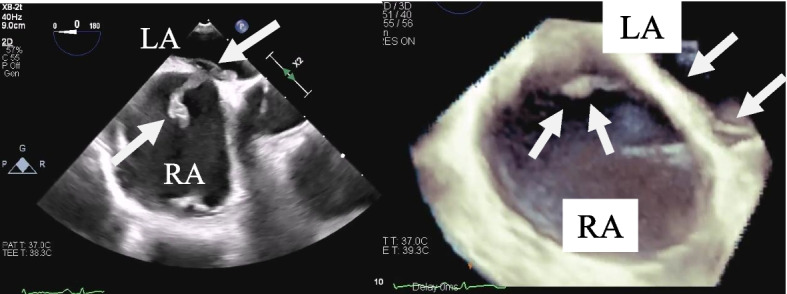
Transesophageal echocardiography showing a thrombus protruding into the left atrium through the patent foramen ovale and fluttering in both atria (arrow: thrombus, RA: right atrium, LA: left atrium)

A 24-year-old woman was admitted to our hospital with chest pain and dyspnea. She was obese, (body mass index: 28.5 kg/m^2^) and had a sedentary lifestyle because of a mental disorder, for which she was taking multiple oral antipsychotic medications and oral contraceptive. Examination revealed tachycardia of approximately 110 beats per minute; systolic blood pressure: 113 mmHg, and percutaneous oxygen saturation: 92%. Blood tests revealed elevated levels of B-type natriuretic peptide (692.4 pg/mL), C-reactive protein (5.0 mg/dL), and D-dimer (11.2 mg/mL). X-rays showed marked cardiomegaly (Fig. 1). Computed tomography confirmed the presence of a massive pulmonary embolism (Fig. 2), and lower limb venous ultrasonography showed a thrombus distal to the left common femoral vein, diagnosed as deep vein thrombosis. Transthoracic echocardiography revealed a rod-shaped mobile thrombus at the foramen ovale, decreased right ventricular function, right ventricular enlargement, and flattened intraventricular septum (Fig. 3). Transesophageal echocardiography was performed to confirm the presence of a mobile thrombus lodged at the foramen ovale, which revealed a large, club-shaped thrombus in both atria penetrating the patent foramen ovale (Fig. 4). We diagnosed IPDE, and because of the risk of sudden arterial embolism, we performed emergency thrombectomy.

**Fig. 5:**
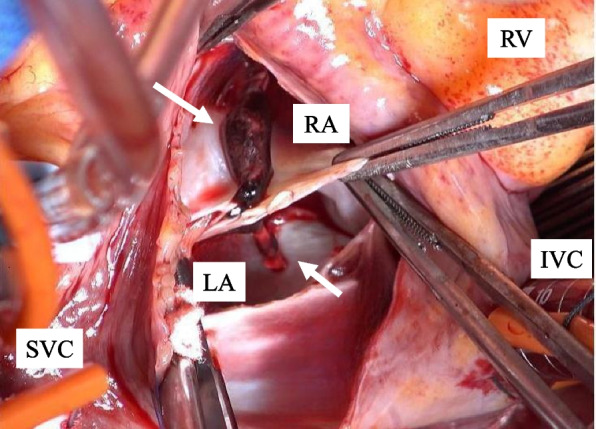
Intraoperative photograph. The fossa ovalis is incised, revealing the interior of both atria. A rod-shaped thrombus is lodged in the patent foramen ovale and protrudes into both atria (arrow: thrombus, RA: right atrium, LA: left atrium, RV: right ventricle, SVC: superior vena cava, IVC: inferior vena cava)

Thrombectomy was performed under cardiopulmonary bypass with ascending aortic cannulation and bicaval venous cannulation through a standard median sternotomy. A rod-shaped thrombus was observed at the foramen ovale from a right atrial incision. Upon incision of the fossa ovale, the thrombus was seen penetrating the patent foramen ovale and protruding into both atria (Fig. 5). The thrombus was removed, and the patent foramen ovale and the incision line on the atrial septum were closed with 5–0 propylene sutures. As a result of a preoperative discussion with the internist based on the computed tomography images, we decided not to perform pulmonary artery thrombectomy owing to the thought that anticoagulant therapy would be sufficient to dissolve the thrombus.

Postoperatively, we did not insert a pulmonary artery catheter due to the potential risk of dislodging the pulmonary artery thrombus; therefore, postoperative management comprised daily echocardiograms performed by the cardiology team. After changing the patient’s position approximately 10 h after surgery, she experienced a sudden decrease in oxygenation capacity, with a percutaneous oxygen saturation of 92% on 100% oxygen and worsening right heart failure. The right ventricle was significantly enlarged and not contracting, which were thought to be caused by the thrombus in the pulmonary artery moving into the peripheral pulmonary arteries. Deep vein thrombosis was considered a possible cause, but no changes were observed in the femoral venous thrombus. Therefore, she was treated for right heart failure with dobutamine, nitric oxide therapy, and anticoagulant therapy with heparin and warfarin. Subsequently, both oxygenation capacity and right heart function gradually improved. Nitric oxide therapy was administered until day 7, and dobutamine until day 8. To prevent recurrence of symptoms due to the residual thrombus in the left lower limb veins as the patient's activity level increases with following rehabilitation, an inferior vena cava filter was placed on postoperative day 8. Thereafter, anticoagulant therapy was performed with edoxaban. She was weaned off the ventilator on postoperative day 9, her oxygenation improved gradually, and oxygen was no longer needed. She was discharged home on postoperative day 34. Two years have passed, and she is asymptomatic, with improved right heart function on echocardiography. Furthermore, the thrombus in her lower extremities had disappeared.

## Discussion

We experienced a case of IPDE complicated by pulmonary embolism, in which a thrombus was incarcerated in a patent foramen ovale, with fluttering in both atria. The patient underwent surgical thrombectomy to avoid arterial embolism.

IPDE is a rare condition characterized by a thrombus straddling a patent foramen ovale, carrying a heightened risk of arterial embolism, and is often associated with high mortality rates with concurrent pulmonary embolism [1]. There are various causes of paradoxical embolism [2], and in this case, it was suspected that deep venous thrombosis had led to pulmonary embolism and right heart failure, with the thrombus traversing the patent foramen ovale owing to elevated right atrial pressure.

There are case reports of surgery performed for IPDE [3, 4], but few reports have described the thrombus penetrating a patent foramen ovale and protruding into both atria, as in this case. Although thrombolytic therapy has been used in the treatment of IPDE [5], systematic review of treatment for IPDE [6] demonstrated that surgical thrombectomy group had lower mortality than thrombolysis treatment or anticoagulation group. Further, there was a non-significant trend toward improved survival and significant reduction of the incidence of systemic embolism in the surgical thrombectomy group compared with anticoagulation group. Therefore, in patients who can tolerate cardiac surgery, surgical thrombectomy is recommended in patients with IPDE. We elected not to remove the pulmonary artery embolism because of thinking that anticoagulation therapy was sufficient to dissolve the thrombus. However, postoperative worsening of pulmonary embolism led to decreased oxygenation capacity and right heart failure. This suggests that pulmonary embolus removal should have been performed.

## Conclusions

Emergency thrombectomy and the patent foramen ovale closure for IPDE were effective in preventing paradoxical embolism. Although pulmonary thromboendarterectomy might have prevented the postoperative deterioration of pulmonary hemodynamics, we experienced a case of postoperative pulmonary embolism that was successfully managed with nitric oxide therapy, inotropes, and anticoagulation, achieving a favorable outcome without the use of mechanical circulatory support.

## Data Availability

Not applicable.

## Abbreviation

IPDE: Impending paradoxical embolism
